# Rich but not poor conditions determine sex‐specific differences in growth rate of juvenile dioecious plants

**DOI:** 10.1007/s10265-021-01296-2

**Published:** 2021-04-16

**Authors:** Kinga Nowak, Marian J. Giertych, Emilia Pers-Kamczyc, Peter A. Thomas, Grzegorz Iszkuło

**Affiliations:** 1 Institute of Dendrology, Polish Academy of Sciences, Kórnik, Poland; 2 Faculty of Biological Sciences, University of Zielona Góra, Zielona Góra, Poland; 3 School of Life Sciences, Keele University, Keele, UK

**Keywords:** Dioecy, Juveniles, *Juniperus communis* - secondary sexual dimorphism, *Taxus baccata*

## Abstract

Causes of secondary sexual dimorphism (SSD) in dioecious plants are very poorly understood, especially in woody plants. SSD is shown mainly in mature plants, but little is known about whether secondary sexual dimorphism can occur in juveniles. It is also assumed that stress conditions intensify differences between the sexes due to the uneven reproductive effort. Therefore, the following research hypotheses were tested: (1) secondary sexual dimorphism will be visible in juveniles; (2) unfavourable soil conditions are the cause of more pronounced differences between the sexes. Rooted shoots of the common yew (*Taxus baccata* L.) and common juniper (*Juniperus communis* L.), previously harvested from parental individuals of known sex were used in the study. During two growing seasons vegetation periods and four times a year, comprehensive morphological features of whole plants were measured. Some SSD traits were visible in the analysed juveniles. Contrary to expectations, differences were more pronounced in the fertilized treatment. Both species reacted to fertilization in different ways. Female yew had a clearly higher total plant mass, root mass, and mean root area when fertilized, whereas male juniper had a higher root mass when fertilized. Differences between the sexes independent of the fertilization treatment were seen, which can be interpreted as sexual adaptations to a continued reproduction. Female yews and male junipers made better use of fertile habitats. Our study showed that SSD may be innate, and sexual compensatory mechanisms could generate uneven growth and development of both sexes. Because the SSD pattern was rather different in both species, it was confirmed that SSD is connected with the specific life histories of specific species rather than a universal strategy of dioecious species.

**Supplementary Information:**

The online version contains supplementary material available at 10.1007/s10265-021-01296-2.

## Introduction

Genders of dioecious plants (with separate male and female individuals) may have different resource allocation due to their different sexual functions. The consequence is secondary sexual dimorphism (SSD) observed in many dioecious species. SSD is often noted in trees, probably due to their longevity and, in contrast to herbaceous species, they grow and reproduce at the same time (Koenig and Knops 1998; Obeso 2002). Resources thus have to be allocated in parallel to growth, maintenance (including defence) and reproduction (Geber et al. 1999).

SSD can be expressed as differences in the growth rate of woody species. Most often, female individuals show a lower growth rate compared to male, due to a greater allocation of resources to reproduction including a longer reproductive effort associated with the process of maturation of seeds and associated structures (Obeso 2002; Bañuelos and Obeso 2004; Montesinos et al. 2006; Chen et al. 2014). However, this is not always clear cut since there are also studies indicating a higher growth rate of female individuals (Xu et al. 2008; Rozas et al. 2009; DeSoto et al. 2016) or a lack of gender differences (Marion and Houle 1996; Rovere et al. 2003).

Most often, SSD is treated as a consequence of sex-specific differences in reproductive effort (Delph and Meagher 1995), however, there are reports that some differences may appear before the organisms reach sexual maturity (Nicotra 1999). These reports are not many, especially in perennial plants, because it is often difficult to visually determine the sex of individuals, until they produce flowers or strobili (Gehring and Linhart 1993).

Gender differences are often most visible in specific, stressful conditions, such as at higher high-altitude sites, in drought conditions or reduced soil fertility (Iszkuło et al. 2011a; Zhao et al. 2011; Huang et al. 2018; Díaz-Barradas et al. 2018; Zhang et al. 2019) which is related to the lower availability of resources. Therefore, SSD may be the result of differences in gender requirements in relation to resources (Barrett and Hough 2013).

In response to resource availability, different genders may have specific compensatory mechanisms (Ueno et al. 2006; Tozawa et al. 2009) allowing better uptake or more efficient use of resources. These include differences in the time spent on reproduction and growth (Delph 1990; Obeso 2002; Milla et al. 2006), photosynthetic capacity (Dawson and Ehleringer 1993; Obeso 2002; Nicotra et al. 2003), or morphological differences such as leaf area (Wallace and Rundel 1979; Meagher 1992; Kohorn 1994; Nowak-Dyjeta et al. 2017). These mechanisms may be of particular importance during masting events (Montesinos et al. 2012).

SSD can lead to Sexual Spatial Segregation (SSS), most often where female individuals are under greater environmental pressure and hence in many species in poorer habitats they show higher mortality or weaker growth (Bierzychudek and Eckhart 1988). As a consequence, sex ratio is often male-biased in dry or less nutrient habitats and female-biased in more favourable conditions (Iglesias and Bell 1989; Nuñez et al. 2008; Garbarino et al. 2015). However, it is not clear whether these gender differences exist prior to reproduction beginning or at times when it is limited, and indeed whether the effect would be clearer in favourable or stressful growing conditions.

It is also unknown if species of different natural habitats will react similarly, as their adaptations and factors limiting growth may be different. Earlier studies on two dioecious species, common yew (*Taxus baccata* L.) and common juniper (*Juniperus communis* L.), showed differences in gender response to environmental resources: juniper growing in N-poor pioneer habitats had a long-term strategy for N storage by females, but yew growing in richer, late successional habitats, did not show such adaptation (Nowak-Dyjeta et al. 2017).

Both these species show certain similarities, they are wind pollinated, zoochoric and evergreen. However, they occupy quite different ecological niches. Yew is a species of fertile, humid habitats and it is shade-tolerant (Thomas and Polwart 2003). In contrast, juniper is a pioneer species inhabiting dry and poor habitats (Thomas et al. 2007). These species also differ in the length of the seed ripening period; in the case of yew, the seeds with arils ripen in the same season as pollination, whereas in juniper the berry-like cones containing the seeds this process takes 2 to 3 years. It can be expected that differences in species ecology and seed ripening time will affect strategies for allocating resources to growth and development.

The following hypotheses were tested: (1) secondary sexual dimorphism will also be visible in juveniles; (2) unfavourable soil conditions are the cause of more pronounced differences between the sexes.

## Materials and methods

The experiment was conducted at the Institute of Dendrology, Polish Academy of Sciences in Kórnik, Poland. Rooted cuttings of *Taxus baccata* and *Juniperus communis* were used in the experiment. In 2012, fifty shoots were collected and rooted from 20 trees of *T. baccata* growing in the Kornik Arboretum, Poland (52°14′40.4″ N 17°06′04.7″ E) and from 20 trees of *J. communis* growing in the Rokita Forest District (Western Pomerania, Poland 53°46′29.8″ N 14°45′24.6″ E). Parent plants had visible reproductive structures (male or female strobili buds and/or female cones). In total, this gave two species × 10 males × 10 females × 50 shoots = 2000 plants. Cuttings of similar size were taken from the middle part of each crown, growing in similar, partially-shaded light conditions. Individuals were grown in 5-L pots under 2-m-high scaffolding with shading net to produce a 50% reduction in full sunlight. The degree of light reduction was confirmed by measurements of relative photosynthetic photon flux density using a line quantum sensor (Apogee Inc.) following the methods of Messier and Puttonen (1995). The soil for the pots was obtained from a natural forest with 10% of the soil volume added from a stand of *T. baccata* or *J. communis* to ensure natural mycorrhizal inoculation. In March 2013, plants were divided into two blocks containing both genders, and then within each block two fertilization treatments were established. Plants from the same paternal or maternal origin were present in both treatment groups with the same number of individuals in each group. The fertilized group of plants received 6 g per L of Osmocote Exact 5–6 M (ICL, Israel) in March 2014 and 2015, whereas non-fertilized plants were grown without any additional fertilizer. The fertilizer contained 15% N, 9% P, 12% K, 2.5% MgO, and microelements. Plants were sampled in each of two years in spring (March) spring (June), autumn (September) and winter (December). The dates of plant collection were associated with the strobili production period (March), the end of new shoots growth (June), the end of yew ripening and the end of vegetative growth, the development of vegetative and generative buds (September), and the dormant period (December). Forty plants from each species were collected at each time (5 plants × 2 sexes × 2 fertilization treatments × 2 blocks). Only in the first two dates of gathering samples (first spring and first summer) were the plants without reproductive organs; from the first autumn and later all cuttings had (or showed signs of having had) reproductive determinants at the time of harvesting. Plants were cleaned of soil, then separated into roots, shoots and needles. Root parameters (mass, length, area and diameters of roots) and needle area were measured using WinSeedle and WinRhizo (Regent Inc.) image analysis systems. Stomatal density (number of stomata per mm^2^) was measured from the mid-point of the abaxial side of the needles with a light microscope and recalculated on a projected needle area basis. Following this, needles, stems and roots were dried at 65 °C for 3 days and weighed to calculate specific leaf area (SLA – leaf area/leaf dry mass, cm^2^ g^− 1^), specific root area (SRA – root area/ root mass, cm^2^ g^− 1^ ), specific root length (SRL – specific root length, cm g^− 1^) and the ratio of root length and root dry mass. The intensity of strobili and cone production in juvenile trees used in the experiment was also determined. For this purpose, the percentage of generative structures in the mass of the shoot and needles from the last two yearly increments was used, because male strobili appear on the current year shoot, female cones on the last (two years old) shoot. Such a comparison was made once in January of the second year of the experiment for males and summer of the second year for females. For comparison, the intensity of reproduction for adults was taken from yews from parallel observations from the Kórnik Arboretum (52°14′27.93″ N; 17°50′28.21″ E) and for junipers from the Torzym population (52°18′36.93″ N; 15°7′43.07″ E) for 10 males and 10 females respectively.

## Statistical analyses

After initial testing of residuals normality (Shapiro–Wilk test) and equal variance (Levene test), a mixed analysis of variance (ANOVA) model with restricted maximum likelihood (REML) was used to evaluate the influence of sex, fertilization, year and season (fixed factors) and their interaction on growth and morphological parameters. Parental tree and block nested within the year were random factors. Mixed analysis of covariance (ANCOVA) was used to compare data of stomata density from different treatment (sex, fertilization, year and season-fixed factors), stomatal row numbers was used as a covariant and parental tree and block as random factors. The results expressed as percentages were arcsin transformed for analyses by ANOVA. The post hoc Tukey test was used to assess the differences among treatments. All analyses were performed using JMP software (version 15.0.0), SAS Institute, Cary, NC, USA.

## Results

### Mass

**Fig. 1:**
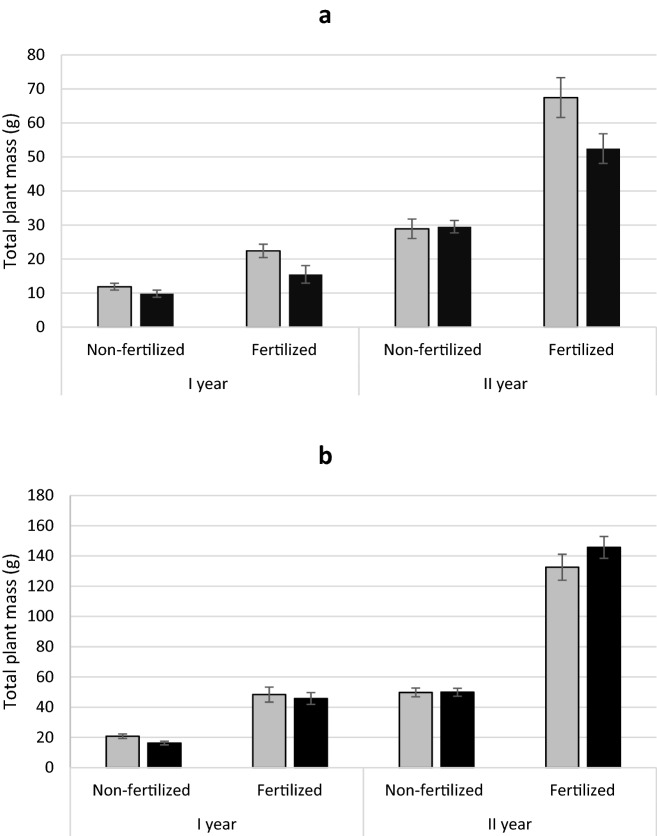
Mean (± standard error) total plant mass of *Taxus baccata* (**a**) and *Juniperus communis* (**b**) individuals. Female grey bars and male black bars

Fertilization significantly affected the total mass of plants of both analysed species (Tables 1, 2), fertilized individuals had a significantly higher mass (Fig. 1 a, b). In the case of yew, a significant interaction between fertilization and sex was demonstrated. Female individuals had a higher mass (mean 45.0 g) than male individuals (mean 34.4 g) in the fertilized treatment (Fig. 1 a). In the non-fertilized treatment, no significant differences in plant mass were observed between the sexes (Fig. 1 a). In the case of juniper, there were no differences between sexes and no interaction between sex and fertilization were found (Table 2; Fig. 1b).

**Table 1:** *Taxus baccata*: diferences in traits between female (F) and male (M), fertilizated (N) and non-fertilized (K) and first (I) and second (II) year observation and season

	Total mass	Root mass	Aboveground mass	Root allocation	Aboveground allocation	Needle area	SLA	Stomatal density	Root area	Fine root to total root area	SRA	SRL
Sex	ns	* F > M	ns	* F > M	** M > F	** F > M	ns	ns	* F > M	ns	* M > F	** M > F
Fertilization	***N > K	*** N > K	*** N > K	*** K > N	*** N > K	*** N > K	* K > N	ns	N > K ***	ns	*** K > N	*** K > N
Sex × fertilization	** FN > MN > FK,MK	** FN > MN > FK,MK	** FN > MN > FK, MK	ns	ns	ns	ns	ns	* FN > MN,FK,MK	ns	ns	ns
Year × sex	ns	ns	ns	ns	ns	ns	ns	ns	ns	ns	* I M > I F > II F, II M	ns
Season × sex	ns	ns	ns	ns	ns	ns	ns	ns	ns	ns	ns	ns

By ANOVA-repeated measures analysis, *P* values < 0.05−0.01 indicate *, *P* = 0.01−0.0001 indicate **, *P* < 0.0001 indicate ***, *ns* not significant

**Table 2:** *Juniperus communis*: diferences in traits between female (F) and male (M), fertilizated (N) and non-fertilized (K) and first (I) and second (II) year observation and season *Sp* Spring, *Sm* Summer, *W *Winter, *Am* Autumn

	Total mass	Root mass	Aboveground mass	Root allocation	Aboveground allocation	Needle area	SLA	Stomatal density	Root area	Fine root to total root area	SRA	SRL
Sex	ns	ns	ns	** M > F	* F > M	*F > M	* M > F	ns	ns	* M > F	ns	ns
Fertilization	*** N > K	*** N > K	*** N > K	*** K > N	*** N > K	ns	ns	ns	*** N > K	*** K > N	ns	*** K > N
Sex × fertilization	ns	* MN > FN > FK,MK	ns	ns	ns	ns	ns	ns	* M > F	* MK > FK ≥ MN > FM	ns	ns
Year × sex	ns	* MII > FII > MI,FI	ns	ns	ns	ns	* IM > IF	ns	*	ns	ns	ns
Season × sex	ns	ns	ns	ns	*	ns	ns	ns	ns	* M_Sp_, F_Sp_>M_W_, F_W_> M_Sm_>F_Sm_>M_Am_>F_Am_	ns	ns

By ANOVA-repeated measures analysis, *P* values < 0,05 − 0,01 indicate *, *P* = 0,01 − 0,0001 indicate **, *P* < 0,0001 indicate ***, *ns* not significant

### Mass allocation

**Fig. 2:**
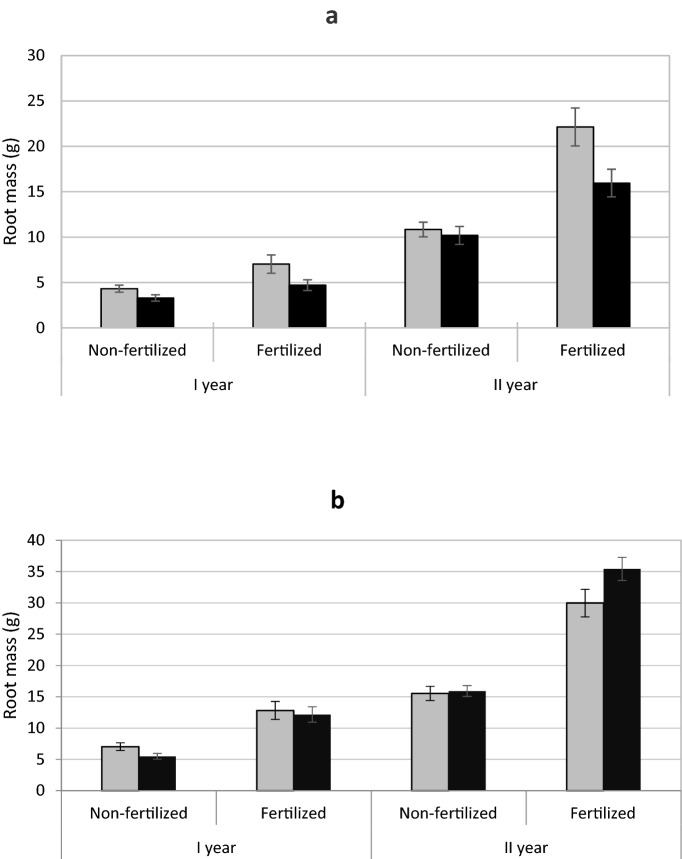
Mean (± standard error) root mass of *Taxus baccata* (**a**) and *Juniperus communis* (**b**) individuals. Female grey bars and male black bars

In yew, the patterns of above-ground and below-ground mass were similar to those for total biomass (Table 1; Fig. 2a; Appendices 1, 2). In juniper, the above-ground mass showed a similar pattern to total mass, however, an interaction of sex and year of harvest was found in below-ground mass. (Table 2). In the first season, the sexes did not differ from each other, but in the second season root mass was higher in male plants (Fig. 2b). There was also interaction between sex and fertilization in juniper (Table 2), such that fertilized male plants had a larger root mass than in females. There was no significant difference between sexes in the unfertilized treatment (Fig. 2b).

**Fig. 3:**
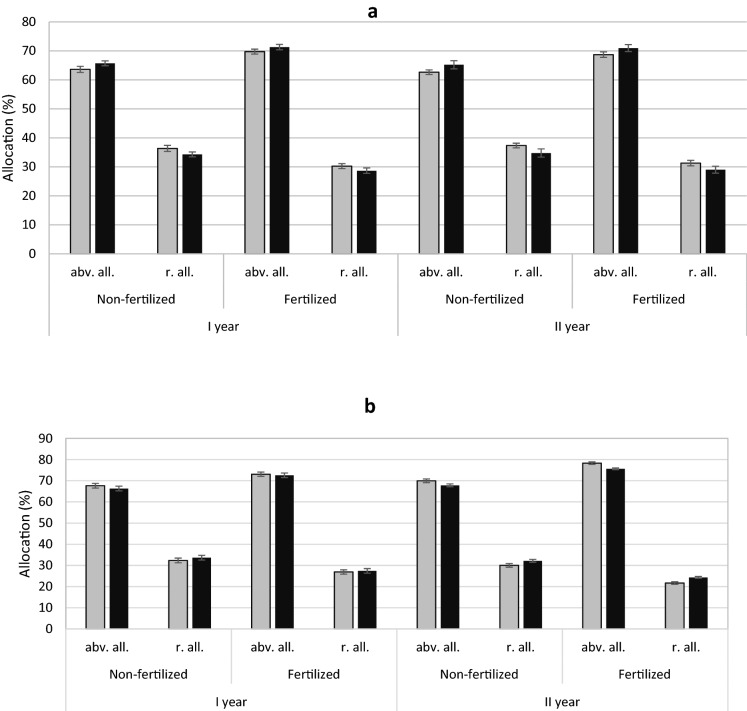
Mean above-ground (abv. all.) and root allocation (r. all.) (± standard error) of *Taxus baccata* (**a**) and *Juniperus communis* (**b**) individuals. Female grey bars and male black bars

In the case of yew, the influence of sex on mass allocation was demonstrated; male individuals had a higher above-ground allocation than female individuals, while female individuals had a higher share of the biomass in roots. This trend was similar in both fertilization treatments (Table 1; Fig. 3a). In contrast, female junipers had a higher above-ground and lower root biomass compared to male specimens (Table 2; Fig. 3b).

### Needle area, stomatal density and SLA

**Fig. 4:**
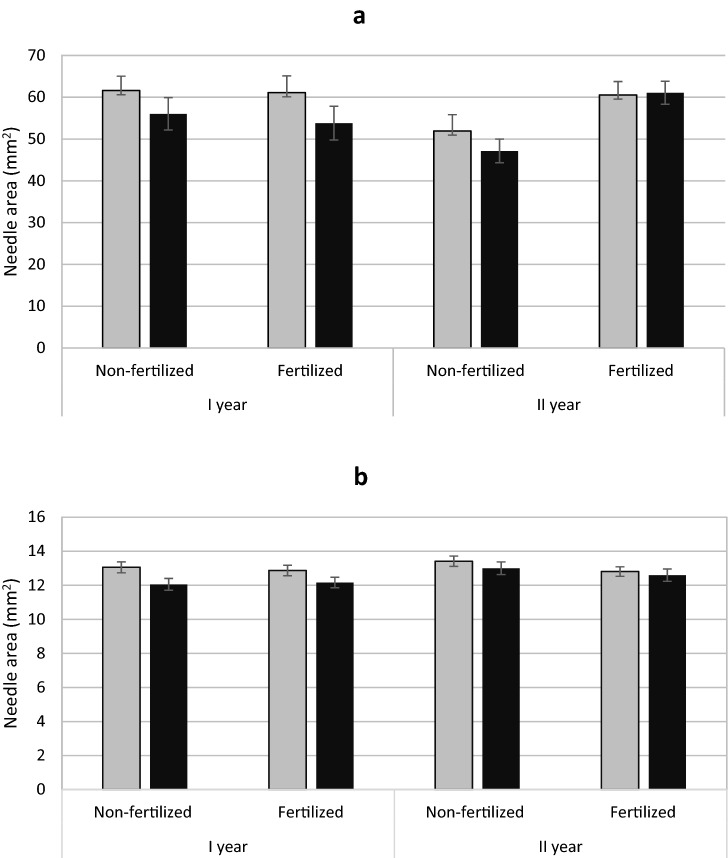
Mean needle area (± standard error) of *Taxus baccata* (**a**) and *Juniperus communis* (**b**) individuals. Female grey bars and male black bars

**Fig. 5:**
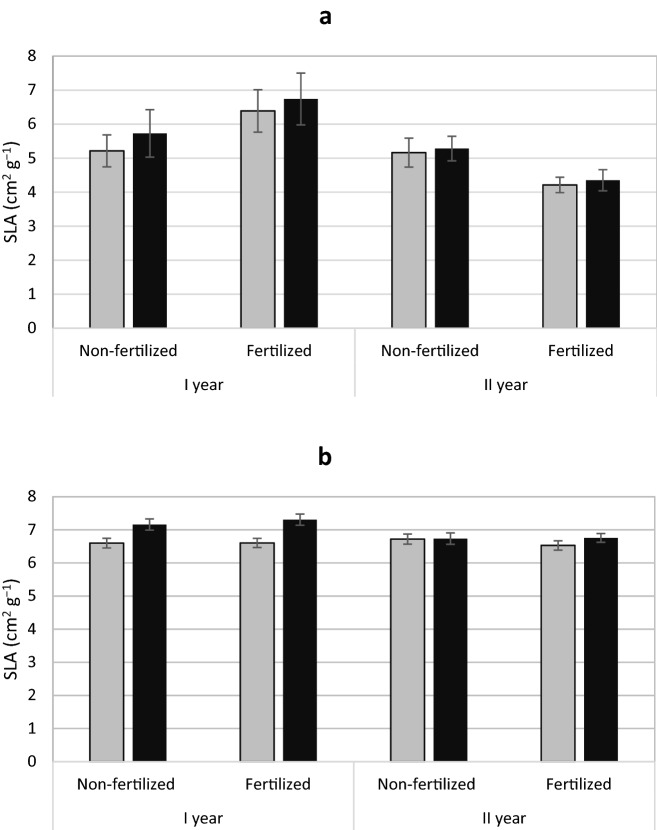
Mean specific leaf area (SLA) ± standard error of *Taxus baccata* (**a**) and *Juniperus communis* (**b**) individuals. Female grey bars and male black bars

Both yew and juniper females had needles with a larger area (Fig. 4a, b) with no interaction of sex × fertilization was found (Tables 1, 2). There was no evidence of gender on SLA of yew. In the case of juniper, male individuals had a slight but significantly higher SLA than females (Table 1; Fig. 5b; Appendix 2). No effect of sex and fertilization on stomatal density was demonstrated (Tables 1, 2; Appendix 1).

### Root area, fine to total root area, SRA and SRL

**Fig. 6:**
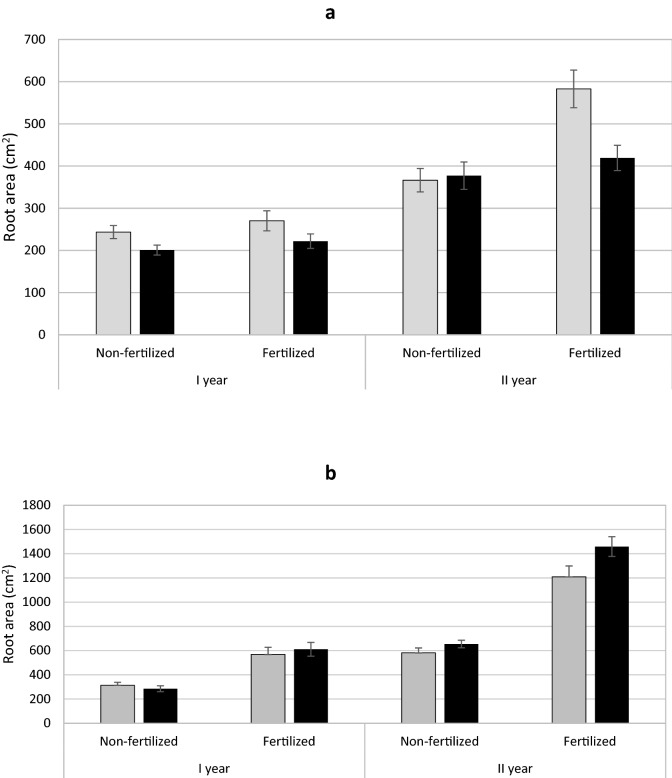
Mean root area (± standard error) of *Taxus baccata* (**a**) and *Juniperus communis* (**b**) individuals. Female grey bars and male black bars

In the case of yew, female individuals had a larger total root area. There was an interaction between sex and fertilization such that female individuals had a larger root area in the fertilized treatment (Table 1; Fig. 6a). Juniper showed no effect of sex on the total root area, however, an interaction between sex and fertilization was demonstrated (Table 2). In the first year, non-fertilized females had a slightly larger root area, but in the second year males had a much greater root area in the fertilized treatment (Fig. 6b).

**Fig. 7:**
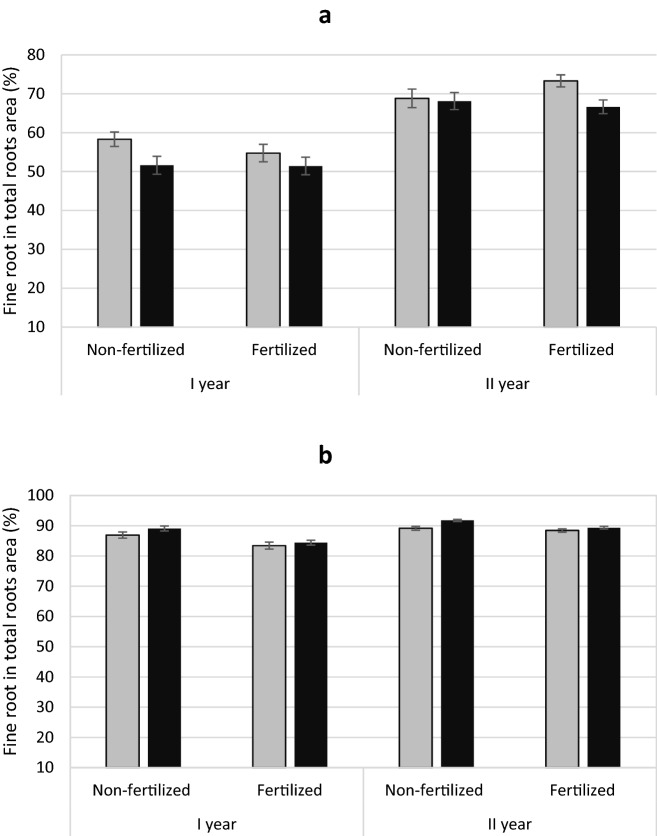
Mean percentage of fine root area in total roots area (± standard error) of *Taxus baccata* (**a**) and *Juniperus communis* (**b**) individuals. Female grey bars and male black bars

**Fig. 8:**
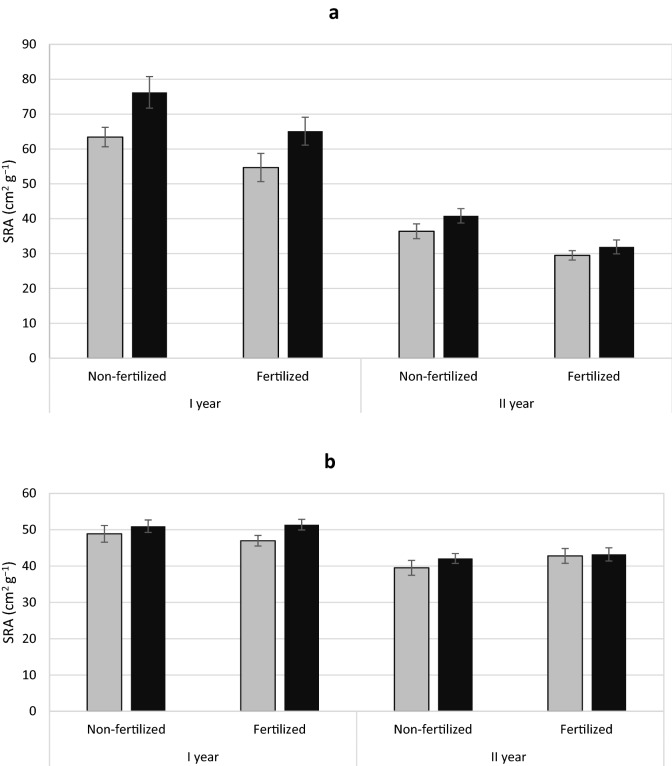
Mean specific root area (SRA) ± standard error of *Taxus baccata* (**a**) and *Juniperus communis* (**b**) individuals. Female grey bars and male black bars

**Fig. 9:**
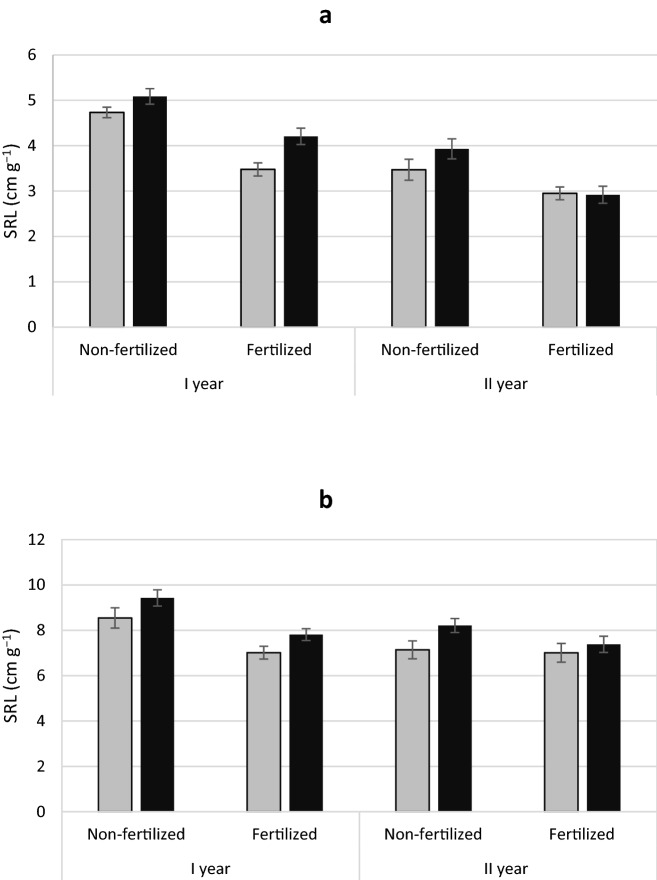
Mean specific root length (SRL) ± standard error of *Taxus baccata* (**a**) and *Juniperus communis* (**b**) individuals. Female grey bars and male black bars

Yew showed no treatment differences in the ratio of fine root area to total root area (Table 1 Fig. 7a). However, male yews had higher specific root area (SRA) and higher specific root length (SRL) than females (Figs. 8a, 9a). In contrast, male junipers had a larger ratio of fine to the total area roots (Table 2; Fig. 7b) but showed no significant differences in SRA or SLR between treatments (Figs. 8b, 9b).

### Pattern of generative structure allocation

A much lower proportion of generative parts was demonstrated for the juvenile plants from the experiment compared to mature plants growing in the field. The proportion of yew male strobili buds in the total mass of the current and last shoot was: in the field 30.14% ± 6.27% (SE), in the experiment 9.62% ± 1.87% (SE). In the case of juniper, the proportion in male plants was: in the field 10.11% ± 1.25% (SE) in the experiment 3.7% ± 1.13% (SE). The proportion of yew female cones in the total mass of the current and last shoot was: in the field: 5.36% ± 1.19% (SE), in the experiment: 2.17% ±0.63%(SE). In the case of female junipers: in the field, 12.45% ± 1.27% (SE) and in the experiment 0.39% ± 0.27% (SE).

## Discussion

In this study we showed that juveniles, similarly to mature trees, exhibit SSD. However, not all sexual differences in the two species have the same pattern in juvenile and mature individuals. Unlike adults (Cedro and Iszkuło 2011), juvenile female yews showed a higher growth rate compared to male ones. In the case of juniper, males had a higher root mass, which indicates a similar growth pattern as in mature plants (Iszkuło and Boratyński 2011). At the same time, it was shown that the area of needles is larger in both species, which is a common feature for mature individuals (Nowak-Dyjeta et al. 2017). A higher growth rate of females compared to males has also been demonstrated in tropical shrubs, *Siparuna grandiflora* (Nicotra 1999) and a common species in the northern hemisphere, *Salix myrsynifolia* (Nybakken and Julkunen-Tiitto 2013). However, it should be mentioned that our study does not ultimately answer the question of whether the traits appear before sexual maturity, because the studied plants came from rooted mature individuals (not from seeds) and saplings started showing reproductive structures very early. From the autumn of the 1st year, all individuals had sexual determinants, although not necessarily at the time of harvest.

It should also be noted that the greater mass in the female yew and male juniper roots was shown only in the fertilized variant. Therefore, we have been rejected the second hypothesis that unfavourable soil conditions are the cause of more pronounced differences between the sexes. This may be due to different investment strategies of both sexes (Galfrascoli and Calviño 2020). Until now, unfavourable habitat conditions were most often considered as a cause of polarization of differences between sexes (see review Retuerto et al. 2018). Under nutrient deficiency, female plants divide resources into growth and reproduction, and consequently often have a lower growth rate. However, plants can regulate the distribution of resources and make vegetative growth a priority with only surplus resources are spent on reproduction (Kozlowski and Wiegert 1986, 1987). Sexual differences in the fertilized variant in our experiment may also be due to the expenditure on reproduction being lower than in mature individuals, which may change the distribution of resources pattern. However, sexual dimorphism in better site conditions has also found in mature individuals of other species. Greater growth of female individuals were obtained for *Acer negundo*. In humid (favourable) conditions, non-reproducing female individuals achieved a higher growth rate compared to male individuals. In dry (unfavourable) conditions, male and female individuals did not differ in growth rate (Dawson and Ehleringer 1993). Similarly, a study of *Populus cathayana* showed a more pronounced growth response of female individuals under high N fertilization. Female individuals showed a higher efficiency in the use of high N (Chen et al. 2014). Juniper did not show significant differences in total biomass of male and female individuals. However, differences were observed for root parameters. In the fertilized treatment, the mass of roots and their area was significantly higher in male junipers. The result for juniper is more typical of better male growth for dioecious species. It should be emphasized, as for yew, the result was only significant in fertilization conditions.

In our experiment, the reproductive allocation element was reduced due to the juvenile phase of plants. In both yew and juniper, the ratio of generative organ to shoot mass was clearly lower in the juvenile plants than in mature. The generative versus vegetative trade-off may have affected the results. Certainly, the removal of inflorescence buds from *Corema abum* (Álvarez-Cansino et al. 2010) produced increased vegetative growth in both sexes. However, the response of female individuals to the reduction of reproduction effort was more pronounced. Thus, the reduction in reproduction costs had a more pronounced effect on female individuals - especially during seed development, which further may confirm the trade-off between vegetative and generative growth costs. A similar pattern of response to reduced reproduction costs was demonstrated in *Acer negundo* where non-reproducing individuals achieved a higher growth rate compared to reproducing individuals (Dawson and Ehleringer 1993).

## Compensatory mechanisms

The results of our study show different mechanisms of compensating reproductive effort in male and female individuals of dioecious species. Particularly noteworthy are those that can occur as innate in juvenile and adult individuals. They indicate that not all secondary dimorphism traits are effects of reproduction on resource balance, but may be genetically determined. They can cause a higher growth rate of individuals of one sex in the early stage of development. As a result, they provide an advantage before later periods of greater reproductive effort, which is a greater burden in female specimens (Nicotra 1999; Nybakken and Julkunen-Tiitto 2013). The lack of such a mechanism in juniper may be due to the adaptation of this species to growth in extremely poor habitats.

Higher growth rate is observed under fertilized conditions. However, in our research, there were differences between the sexes in some features (e.g. biomass allocation in the aboveground mass and roots, as well as specific features of the roots such as SRA, SRL) regardless of fertilization. These adaptations or compensation mechanisms, are associated with the uptake or use of habitat resources. They are more often found in female individuals with high reproduction costs (Dawson and Geber 1999; Obeso 2002; Barrett and Hough 2013). These mechanisms may allow the diversion of more resources to growth in more favourable conditions as demonstrated in yew. Nevertheless, these mechanisms may function regardless of habitat conditions, as seen by the larger needle area of yew and juniper females regardless of fertilization. This is a common feature in female individuals of dioecious species (Wallace and Rundel 1979; Meagher 1992; Kohorn 1994), and has previously been demonstrated in adult yew and juniper individuals (Iszkuło et al. 2009, 2011b; Nowak-Dyjeta et al. 2017). A larger needle surface may be associated with greater gas exchange and photosynthesis activity (Dawson and Bliss 1989; Dawson and Ehleringer 1993) increasing the growth of females and allowing them to achieve better growth in favourable conditions such as fertilization. On the other hand, a larger area of needles may be associated with greater sensitivity to lighting. Photochemistry studies in the same experiment showed that female yew were more threatened by photoinhibition when exposed to high light (Robakowski et al. 2018). Such morphological and physiological features indicate better adaptation of female individuals to greater shading. The ability of female to make more effective use of richer habitats was indicated by studies in natural yew conditions in Italy, where female individuals were more often found in better habitat conditions compared to male individuals (Vessella et al. 2015). The higher SLA values found in male juniper individuals may suggest the existence of some adaptation, which, however, is not reflected in plant biomass. Higher SLA is associated with higher photosynthesis intensity per leaf mass unit (Poorter and Evans 1998). This feature is very variable both within the species and even one individual (Reich et al. 1998), and is often equated with the plant’s response to light conditions; low SLA is characterized by leaves in high light intensity (Björkman 1981), and in our experience the lighting conditions were even.

Another feature associated with sex and occurring regardless of fertilization was the allocation to aboveground and underground biomass. In yew there was a greater allocation of resources to the roots in females, and in juniper, inversely to the roots of males. Larger allocation to roots may allow greater uptake of minerals from the substrate (Hermans et al. 2006), directly improving vegetative growth. In the juvenile plants used in this study, resources collected by the roots may be directed to vegetative tissues resulting in better growth. This appears to be case in yew, where females with greater root allocation showed greater overall biomass. Greater allocation of female to roots was also found in *Populus cathayana* (Zhang et al. 2014; Dong et al. 2017). What’s more, under stress associated with a shortage of minerals, the allocation to the roots of female individuals increased due to the reduction in above-ground mass, but not underground. In our studies such a situation was not observed and regardless of the fertility of the substrate, the allocation showed the same sexual pattern.

Roots also play the role of resource storage and, in the case of female individuals, may be important in supporting reproductive effort to provide resources for masting events. Our research was conducted on reproducing juvenile plants but we expect that some of mechanisms and adaptations are still operational even when reproduction is absent or lower, and resource use in females may be more pronounced because of future reproductive effort. Hence, female yews had a lower SRL compared to males, while allocating more biomass to the roots. Lowest SRL occurred in the fertilized treatment, which is in line with the general formula for this trait (Ostonen et al. 2007). However, male individuals have higher SRL values in both fertilization variants, which is only visible in the first observation season. Male individuals with a higher SRL have greater opportunities for soil exploration, but such roots are less durable (Kramer-Walter et al. 2016). Perhaps it is a strategy that allows more nitrogen foraging, which is an important aspect for male specimens of wind-pollinated species, producing large amounts of nitrogen-rich pollen in a short fragment of the growing season creating a high nitrogen demand (Obeso 2002; Nowak-Dyjeta et al. 2017).

In the case of juniper, male individuals showed a greater root allocation, but this had no clear effect in increasing total plant mass regardless of fertilization. However, a larger mass of roots of males in the second year was seen, which should lead to a larger mass of whole plants in subsequent years. Perhaps the differences resulting from this adaptation require a longer observation period to see clear effects. We are aware that the all observed root reactions may be affected by all roots being adventitious, formed during rooting of cuttings.

### Intraspecies differences

Equivocal results are often obtained in SSD research on dioecious species (Retuerto et al. 2018). It can be seen, therefore, that dioecious species often do not react according to one universal pattern and further research is needed into the causes and mechanisms associated with sexual dimorphism. When considering gender differences in different environments, we often observe sexual sex segregation (SSS) and an increase in the proportion of female along a fertility gradient (Bierzychudek and Eckhart 1988; Agren et al. 1999; Dawson and Geber 1999; Dudley 2006; Nuñez et al. 2008). This phenomenon is explained by greater mortality and poorer female vitality in a poor environment, due to higher reproduction resource demands. Our research adds to this issue the claim that SSS may be associated with better adaptation of female individuals (in the case of yew) to fertile environments and better use of soil potential. This corresponds to the observation of a greater growth rate of female yew individuals, while focusing on niches with favourable environmental conditions (Garbarino et al. 2015).

In the case of juniper, males have a higher underground mass allocation and higher SLA, which may indicate a better use of resources compared to female individuals. Generally females juniper are recognized to have greater cost reproduction (Ortiz et al. 2002) and lower growth rate than males (Massei et al. 2006). Perhaps it is an adaptation to more efficient nitrogen acquisition. Male reproductive structures are reported to require more nitrogen than females (McDowell et al. 2000). Juniper is a species of poor habitat and nitrogen capture may be key to successful pollen production. Additionally, our previous studies showed that female junipers are less exposed to the effects of reproductive effort due to the long-term (2–3 years) maturing of cones (Thomas et al. 2007; Nowak-Dyjeta et al. 2017) and have not developed compensatory mechanisms like the yew. Other studies on *Juniperus communis* have also found no differences in growth, environmental sensitivity or microhabitat segregation of sexes (Marion and Houle 1996; Houle and Duchesne 1999; Ortiz 2002).

Juniper as a pioneer species associated with a poor habitat probably manages resources in a different way, possibly by storing resources between years for masting events (Montesinos et al. 2012). However, differences in root mass appearing in the second year may suggest that in future growth male specimens could achieve higher growth parameters.

In this study we showed that juveniles, similarly to mature individuals, exhibit SSD. However, not all sexual differences in the two species have the same pattern in juvenile and mature individuals. In conclusion, in our research, juvenile plants have some SSD traits that may be innate. Sexes show differences in growth traits, and these are much more pronounced in yew. However, these differences are observed in favourable conditions, which was not as expected. It is influenced by the reduction of reproduction costs, which allows the effects of compensation mechanisms to be seen. Both species showed opposite sexual responses to fertilization. Sex differences in growth are more pronounced in the case of yew than juniper. Yew is a species in which female individuals are better adapted to use the soil fertility potential, in the case of juniper, male individuals appear to be better adapted.

## Supplementary Information

Below is the link to the electronic supplementary material.

Supplementary material 1 (DOCX 41.9 kb)
