# Supplementary material for: Rich but not poor conditions determine sex‐specific differences in growth rate of juvenile dioecious plants
Source: J Plant Res. 2021 Apr 16;134(5):947–62. doi: 10.1007/s10265-021-01296-2 (PMC8364908; doi:10.1007/s10265-021-01296-2)
Supplement: Supplementary file 1 — Supplementary material 1 (DOCX 41.9 kb) [file 10265_2021_1296_MOESM1_ESM.docx]

**Electronic supplementary materials**

**Title:** Rich but not poor conditions determine sex-specific differences in growth rate of juvenile dioecious plants

**Authors:** Nowak Kinga, Giertych Marian J., Pers-Kamczyc Emilia, Thomas Peter A., Iszkuło Grzegorz

**Journal:**

Journal of Plant Research

**Corresponding author:** Nowak Kinga, ORCID ID [0000-0001-7037-8349](https://orcid.org/0000-0001-7037-8349),

Institute of Dendrology, Polish Academy of Sciences, Kórnik, Poland,

phone numer: +48 613072423 fax numer: +48 618170166 e-mail: [knd@man.poznan.pl](mailto:knd@man.poznan.pl)

**Content:**

**Tables S1–S2**

**Table S1**: ***Taxus baccata*** and ***Juniperus communis*** full factorial ANOVA results for: total plant mass, aboveground mass, root mass, aboveground allocation and root allocation, needle area, SLA, stomatal density, total roots area, fine root area, fine root length, percentage of fine root area in total root area, SRA, SRL.

|  |  |  | ***Taxus baccata*** | | ***Juniperus communis*** | |
| --- | --- | --- | --- | --- | --- | --- |
| **Parametr** | **effect** | **Df** | **F** | **P** | **F** | **P** |
| **Total mass** | sex | 1 | 2,07 | 0,1516 | 0,1177 | 0,7359 |
|  | fertilization | 1 | 150,48 | <0,0001 | 785,99 | <0,0001 |
|  | sex*fertilization | 1 | 10,01 | 0,0017 | 1,8978 | 0,1696 |
|  | year | 1 | 204,4 | <0,0001 | 799,57 | <0,0001 |
|  | season | 3 | 42,678 | <0,0001 | 80,862 | <0,0001 |
|  | year*sex | 1 | 0,011 | 0,9166 | 2,2604 | 0,1344 |
|  | season*sex | 3 | 0,5905 | 0,6217 | 0,7496 | 0,5235 |
| **Parametr** | **effect** | **Df** | **F** | **P** | **F** | **P** |
| **Aboveground mass** | sex | 1 | 1,442 | 0,2313 | 0,0206 | 0,8876 |
|  | fertilization | 1 | 182,0527 | <0,0001 | 907,73 | <0,0001 |
|  | sex*fertilization | 1 | 10,8802 | 0,001 | 1,3888 | 0,2397 |
|  | year | 1 | 156,1049 | <0,0001 | 1034,7 | <0,0001 |
|  | season | 3 | 37,629 | <0,0001 | 77,370 | <0,0001 |
|  | year*sex | 1 | 0,0133 | 0,9083 | 1,6439 | 0,2016 |
|  | season*sex | 3 | 0,3859 | 0,7633 | 0,7798 | 0,5061 |
| **Parametr** | **effect** | **Df** | **F** | **P** | **F** | **P** |
| **Root mass** | sex | 1 | 5,2037 | 0,0236 | 0,5119 | 0,4844 |
|  | fertilization | 1 | 83,5677 | <0,0001 | 370,75 | <0,0001 |
|  | sex*fertilization | 1 | 10,3634 | 0,0015 | 6,2509 | 0,013 |
|  | year | 1 | 208,1932 | <0,0001 | 521,91 | <0,0001 |
|  | season | 3 | 45,1333 | <0,0001 | 79,697 | <0,0001 |
|  | year*sex | 1 | 0,2705 | 0,6035 | 4,7945 | 0,0296 |
|  | season*sex | 3 | 1,5141 | 0,2112 | 0,9568 | 0,4136 |
| **Parametr** | **effect** | **Df** | **F** | **P** | **F** | **P** |
| **Aboveground allocation** | sex | 1 | 7,0697 | 0,0087 | 6,4554 | 0,0237 |
|  | fertilization | 1 | 96,0676 | <0,0001 | 31,0118 | <0,0001 |
|  | sex*fertilization | 1 | 0,1436 | 0,7051 | 0,0003 | 0,9858 |
|  | year | 1 | 0,0782 | 0,7821 | 1,5104 | 0,3303 |
|  | season | 3 | 22,5293 | <0,0001 | 2,2149 | 0,0869 |
|  | year*sex | 1 | 1,0379 | 0,3096 | 0,4833 | 0,4889 |
|  | season*sex | 3 | 0,6556 | 0,5801 | 2,7451 | 0,0436 |
| **Parametr** | **effect** | **Df** | **F** | **P** | **F** | **P** |
| **Root allocation** | sex | 1 | 5,0117 | 0,0267 | 11,542 | 0,0008 |
|  | fertilization | 1 | 105,5621 | <0,0001 | 174,20 | <0,0001 |
|  | sex*fertilization | 1 | 0,0243 | 0,8762 | 0,0067 | 0,9349 |
|  | year | 1 | 0,263 | 0,617 | 45,967 | 0,0226 |
|  | season | 3 | 25,4334 | <0,0001 | 31,794 | <0,0001 |
|  | year*sex | 1 | 0,4199 | 0,5178 | 3,0691 | 0,0809 |
|  | season*sex | 3 | 0,2249 | 0,879 | 1,535 | 0,2058 |
| **Parametr** | **effect** | **Df** | **F** | **P** | **F** | **P** |
| **Needle area** | sex | 1 | 7,1948 | 0,0081 | 6,1435 | 0,0138 |
|  | fertilization | 1 | 16,042 | <0,0001 | 1,1823 | 0,2779 |
|  | sex*fertilization | 1 | 1,0252 | 0,3123 | 0,1501 | 0,6987 |
|  | year | 1 | 1,1134 | 0,3419 | 17,510 | 0,0545 |
|  | season | 3 | 5,9641 | 0,0006 | 2,2495 | 0,0829 |
|  | year*sex | 1 | 1,2769 | 0,2599 | 1,6653 | 0,198 |
|  | season*sex | 3 | 1,5378 | 0,2052 | 0,6828 | 0,532 |
| **Parametr** | **effect** | **Df** | **F** | **P** | F | P |
| **SLA** | sex | 1 | 0,8954 | 0,3455 | 4,7907 | 0,0454 |
|  | fertilization | 1 | 5,6187 | 0,0185 | 0,0676 | 0,7951 |
|  | sex*fertilization | 1 | 0,01 | 0,9206 | 0,6306 | 0,4279 |
|  | year | 1 | 14,9246 | 0,0463 | 1,0148 | 0,3554 |
|  | season | 3 | 14,4765 | <0,0001 | 38,2213 | <0,0001 |
|  | year*sex | 1 | 2,6868 | 0,1028 | 4,3374 | 0,0395 |
|  | season*sex | 3 | 1,8094 | 0,146 | 2,0317 | 0,1098 |
| **Parametr** | **effect** | **Df** | **F** | **P** | F | P |
| **Stomatal density** | sex | 1 | 0,0123 | 0,9122 | 0,51 | 0,4795 |
|  | fertilization | 1 | 0,3272 | 0,5705 | 3,5422 | 0,0672 |
|  | sex*fertilization | 1 | 2,1589 | 0,1498 | 0,0457 | 0,8318 |
|  | year | 1 | 0,0014 | 0,9756 | 5,3154 | 0,0266 |
|  | year*sex | 1 | 0,0466 | 0,8303 | 0,0111 | 0,9167 |
| **Parametr** | **effect** | **Df** | **F** | **P** | **F** | **P** |
| **Total root area** | sex | 1 | 4,6973 | 0,0313 | 1,1284 | 0,3041 |
|  | fertilization | 1 | 21,2643 | <0,0001 | 281,29 | <0,0001 |
|  | sex*fertilization | 1 | 5,8275 | 0,0165 | 4,5494 | 0,0339 |
|  | year | 1 | 121,410 | <0,0001 | 125,14 | 0,0003 |
|  | season | 3 | 21,565 | <0,0001 | 24,005 | <0,0001 |
|  | year*sex | 1 | 0,0389 | 0,8439 | 2,8361 | 0,0939 |
|  | season*sex | 3 | 0,7098 | 0,5471 | 1,7787 | 0,1515 |
| **Parametr** | **effect** | **Df** | **F** | **P** | **F** | **P** |
| **Fine root area** | sex | 1 | 3,8743 | 0,0504 | 0,247 | 0,247 |
|  | fertilization | 1 | 12,3252 | 0,0005 | <0,0001 | <0,0001 |
|  | sex*fertilization | 1 | 5,6869 | 0,0178 | 0,0438 | 0,0438 |
|  | year | 1 | 113,47 | <0,0001 | 0,0012 | 0,0012 |
|  | season | 3 | 18,8227 | <0,0001 | <0,0001 | <0,0001 |
|  | year*sex | 1 | 0,0993 | 0,7529 | 0,0967 | 0,0967 |
|  | season*sex | 3 | 1,0004 | 0,3914 | 0,2275 | 0,2275 |
| **Parametr** | **effect** | **Df** | **F** | **P** | **F** | **P** |
| **Fine root length** | sex | 1 | 2,8167 | 0,0948 | 2,2043 | 0,1571 |
|  | fertilization | 1 | 8,0868 | 0,0048 | 174,68 | <0,0001 |
|  | sex*fertilization | 1 | 5,3037 | 0,0221 | 3,6468 | 0,0573 |
|  | year | 1 | 91,512 | <0,0001 | 82,019 | 0,0033 |
|  | season | 3 | 17,420 | <0,0001 | 19,995 | <0,0001 |
|  | year*sex | 1 | 0,2165 | 0,6422 | 3,6563 | 0,0575 |
|  | season*sex | 3 | 0,9976 | 0,3945 | 1,0238 | 0,3825 |
| **Parametr** | **effect** | **Df** | **F** | **P** | **F** | **P** |
| **Percentage of fine root area in total root area** | sex | 1 | 1,7747 | 0,1843 | 6,4491 | 0,0228 |
|  | fertilization | 1 | 0,0195 | 0,8891 | 56,7353 | <0,0001 |
|  | sex*fertilization | 1 | 0,3801 | 0,5381 | 4,1072 | 0,0437 |
|  | year | 1 | 57,776 | 0,0007 | 11,3991 | 0,0653 |
|  | season | 3 | 22,717 | <0,0001 | 40,2541 | <0,0001 |
|  | year*sex | 1 | 2,8912 | 0,0904 | 0,5795 | 0,4483 |
|  | season*sex | 3 | 0,7235 | 0,5387 | 2,8925 | 0,0359 |
| **Parametr** | **effect** | **Df** | **F** | **P** | **F** | **P** |
| **SRA** | sex | 1 | 5,903 | 0,0164 | 0,3475 | 0,5634 |
|  | fertilization | 1 | 31,040 | <0,0001 | 0,3036 | 0,5821 |
|  | sex*fertilization | 1 | 0,1741 | 0,6768 | 0,0002 | 0,9882 |
|  | year | 1 | 140,62 | 0,0022 | 12,185 | 0,0245 |
|  | season | 3 | 59,980 | <0,0001 | 39,2939 | <0,0001 |
|  | year*sex | 1 | 4,4514 | 0,0362 | 1,1922 | 0,2763 |
|  | season*sex | 3 | 1,3599 | 0,2555 | 0,7101 | 0,5467 |
| **Parametr** | **effect** | **Df** | **F** | **P** | **F** | **P** |
| **SRL** | sex | 1 | 8,7204 | 0,0037 | 1,5712 | 0,2271 |
|  | fertilization | 1 | 86,559 | <0,0001 | 30,167 | <0,0001 |
|  | sex*fertilization | 1 | 0,0052 | 0,9424 | 1,1370 | 0,2873 |
|  | year | 1 | 43,213 | 0,0137 | 2,7089 | 0,2064 |
|  | season | 3 | 23,740 | <0,0001 | 33,030 | <0,0001 |
|  | year*sex | 1 | 2,4878 | 0,1163 | 0,3762 | 0,5404 |
|  | season*sex | 3 | 2,0723 | 0,1044 | 1,1363 | 0,3348 |

**Table S2:** Mean and standard error of analysed features for *Taxus baccata* L. and *Juniperus communis* L.

| Species | Year | Season | Sex | Fertilization | **Total mass (g)** | **Aboveground mass (g)** | **Root mass (g)** | **Aboveground allocation (%)** | **Root allocation (%)** | **Needle area (mm^2^)** | **SLA (cm2 g^-1^)** | **Root area (cm^2^)** | **Fine roots/total roots area (%)** | **SRA (cm2 g-^1^)** | **SRL (cm g ^-1^)** |
| --- | --- | --- | --- | --- | --- | --- | --- | --- | --- | --- | --- | --- | --- | --- | --- |
| *Taxus baccata* L. | I | Spring | F | K | 6,93 ± 0,86 | 3,97 ± 0,46 | 2,96 ± 0,41 | 58,19 ± 1,21 | 41,81 ± 1,21 | 35,47 ± 3,54 | 8,38 ± 1,32 | 177,95 ± 11,59 | 49,85 ± 3,19 | 68,46 ± 7,35 | 4,45 ± 0,25 |
|  |  |  |  | N | 5,89 ± 0,63 | 4,02 ± 0,43 | 1,87 ± 0,22 | 68,41 ± 1,16 | 31,59 ± 1,16 | 23,5 ± 1,43 | 12,12 ± 1,33 | 136,35 ± 4,96 | 37,76 ± 2,03 | 79,95 ± 7,11 | 3,67 ± 0,23 |
|  |  |  | M | K | 3,47 ± 0,38 | 2,12 ± 0,25 | 1,34 ± 0,15 | 61,17 ± 1,13 | 38,83 ± 1,13 | 23,05 ± 3,86 | 10,92 ± 1,83 | 126,56 ± 4,23 | 34,12 ± 1,82 | 102,89 ± 9,22 | 4,78 ± 0,36 |
|  |  |  |  | N | 5,65 ± 0,64 | 3,92 ± 0,5 | 1,74 ± 0,19 | 68,66 ± 1,77 | 31,34 ± 1,77 | 22,28 ± 2,4 | 13,58 ± 1,38 | 134,69 ± 6,23 | 37,45 ± 2,71 | 82,9 ± 5,89 | 4,06 ± 0,28 |
|  |  | Summer | F | K | 8,1 ± 0,82 | 5,79 ± 0,73 | 2,31 ± 0,19 | 70,02 ± 2,52 | 29,98 ± 2,52 | 76,07 ± 5,1 | 3,48 ± 0,34 | 175,26 ± 8,82 | 48,89 ± 2 | 77,08 ± 2,44 | 4,9 ± 0,19 |
|  |  |  |  | N | 14,38 ± 1,79 | 10,97 ± 1,33 | 3,41 ± 0,48 | 76,29 ± 0,79 | 23,71 ± 0,79 | 68,09 ± 5,05 | 5,01 ± 0,25 | 207,4 ± 13,71 | 52,8 ± 2,28 | 67,04 ± 5,49 | 4,34 ± 0,16 |
|  |  |  | M | K | 8,99 ± 1,7 | 6,04 ± 1,12 | 2,95 ± 0,6 | 67,71 ± 1,39 | 32,29 ± 1,39 | 63,66 ± 6,9 | 3,38 ± 0,37 | 198,59 ± 22,9 | 50,75 ± 3,82 | 80,14 ± 8,67 | 5,3 ± 0,34 |
|  |  |  |  | N | 13,53 ± 3,09 | 10,42 ± 2,42 | 3,11 ± 0,68 | 77,03 ± 1,1 | 22,97 ± 1,1 | 66,85 ± 7,28 | 4,18 ± 0,46 | 195,36 ± 21,7 | 48,9 ± 3,3 | 75,9 ± 7,66 | 4,69 ± 0,27 |
|  |  | Autumn | F | K | 13,85 ± 1,9 | 8,89 ± 1,27 | 4,96 ± 0,76 | 64,35 ± 1,98 | 35,65 ± 1,98 | 64,18 ± 6,22 | 4,78 ± 0,64 | 279,99 ± 27,45 | 62,23 ± 2,74 | 60,92 ± 3,52 | 4,97 ± 0,27 |
|  |  |  |  | N | 30,47 ± 5,02 | 20,35 ± 3,22 | 10,12 ± 1,85 | 67,11 ± 1,2 | 32,89 ± 1,2 | 69,05 ± 4,23 | 4,75 ± 0,42 | 342,75 ± 46,43 | 59,87 ± 4,24 | 36,97 ± 3,46 | 2,84 ± 0,31 |
|  |  |  | M | K | 9,41 ± 0,89 | 6,26 ± 0,6 | 3,15 ± 0,35 | 66,55 ± 1,95 | 33,45 ± 1,95 | 57,34 ± 6,88 | 6,2 ± 1,76 | 210,91 ± 13,6 | 56,42 ± 2,59 | 71,2 ± 4,99 | 5,43 ± 0,33 |
|  |  |  |  | N | 14,09 ± 2,11 | 9,83 ± 1,41 | 4,26 ± 0,74 | 70,21 ± 1,38 | 29,79 ± 1,38 | 56,97 ± 3,63 | 4,94 ± 0,54 | 231,52 ± 27,8 | 54,97 ± 3,73 | 61,13 ± 6,18 | 4,46 ± 0,46 |
|  |  | Winter | F | K | 19,24 ± 1,55 | 11,91 ± 0,99 | 7,34 ± 0,59 | 61,86 ± 0,66 | 38,14 ± 0,66 | 68,44 ± 5,66 | 4,35 ± 0,64 | 343,76 ± 29,75 | 70,14 ± 1,52 | 46,89 ± 1,39 | 4,62 ± 0,18 |
|  |  |  |  | N | 40,21 ± 6,22 | 27,02 ± 4,12 | 13,19 ± 2,22 | 67 ± 1,79 | 33 ± 1,79 | 80,74 ± 5,3 | 4,17 ± 0,37 | 401,35 ± 50 | 67,37 ± 2,84 | 33,02 ± 2,52 | 3 ± 0,18 |
|  |  |  | M | K | 17,4 ± 2,07 | 11,69 ± 1,39 | 5,7 ± 0,74 | 67,38 ± 1,5 | 32,62 ± 1,5 | 63,62 ± 5,16 | 5,01 ± 0,5 | 267,53 ± 23,79 | 65,18 ± 3,22 | 50,72 ± 3,78 | 4,83 ± 0,35 |
|  |  |  |  | N | 30,12 ± 3,08 | 21,18 ± 2,53 | 8,95 ± 0,77 | 69,16 ± 2,01 | 30,84 ± 2,01 | 72,31 ± 6,28 | 3,98 ± 0,31 | 337,43 ± 40,3 | 65,84 ± 3,01 | 37,73 ± 2,85 | 3,56 ± 0,34 |
|  | II | Spring | F | K | 21,75 ± 1,87 | 13,56 ± 1,15 | 8,19 ± 0,74 | 62,45 ± 0,73 | 37,55 ± 0,73 | 79,05 ± 8,85 | 2,79 ± 0,24 | 341,69 ± 23,42 | 71,21 ± 2,57 | 42,69 ± 2,07 | 4,2 ± 0,2 |
|  |  |  |  | N | 36,68 ± 5,33 | 24,55 ± 3,46 | 12,13 ± 1,95 | 67,23 ± 1,42 | 32,77 ± 1,42 | 84,03 ± 6,82 | 3,05 ± 0,3 | 394,16 ± 45,04 | 70,35 ± 1,96 | 34,53 ± 2,36 | 3,26 ± 0,22 |
|  |  |  | M | K | 16,43 ± 1,89 | 10,42 ± 1,14 | 6 ± 0,8 | 63,79 ± 1,46 | 36,21 ± 1,46 | 56,33 ± 4,88 | 4,5 ± 0,5 | 288,77 ± 27,68 | 66,99 ± 2,85 | 49,92 ± 1,94 | 4,7 ± 0,2 |
|  |  |  |  | N | 34,57 ± 6,79 | 24,08 ± 5,21 | 10,49 ± 1,81 | 69,12 ± 2,27 | 30,88 ± 2,27 | 75,63 ± 3,57 | 3,25 ± 0,19 | 411,93 ± 54,42 | 70,48 ± 2,81 | 42,85 ± 2,69 | 4,1 ± 0,17 |
|  |  | Summer | F | K | 18,27 ± 5,59 | 11,6 ± 3,47 | 6,66 ± 2,12 | 63,79 ± 0,79 | 36,21 ± 0,79 | 29,89 ± 4,46 | 8,5 ± 1,3 | 306,23 ± 69,72 | 63,66 ± 5,54 | 51,14 ± 4,89 | 4,26 ± 0,2 |
|  |  |  |  | N | 66,49 ± 16,33 | 50,05 ± 12,53 | 16,44 ± 3,88 | 75,6 ± 1,69 | 24,4 ± 1,69 | 57,35 ± 2,45 | 5,31 ± 0,71 | 520,42 ± 101,46 | 70,9 ± 6,77 | 33,59 ± 1,78 | 3,21 ± 0,29 |
|  |  |  | M | K | 21,03 ± 5,79 | 14,28 ± 3,39 | 6,75 ± 2,58 | 69,75 ± 2,99 | 30,25 ± 2,99 | 33,89 ± 3,3 | 6,75 ± 1,4 | 290,39 ± 82,49 | 60,64 ± 5,58 | 48,93 ± 5,04 | 3,99 ± 0,27 |
|  |  |  |  | N | 33,84 ± 8,01 | 26,24 ± 6,3 | 7,6 ± 1,86 | 77,79 ± 1,73 | 22,21 ± 1,73 | 53,14 ± 5,86 | 4,63 ± 0,82 | 280,83 ± 53,31 | 58 ± 5,04 | 39,81 ± 3,47 | 3,03 ± 0,15 |
|  |  | Autumn | F | K | 35,49 ± 3,22 | 22,52 ± 2,29 | 12,98 ± 1 | 63 ± 1,05 | 37 ± 1,05 | 47,8 ± 4,01 | 5,03 ± 0,37 | 485,35 ± 51,47 | 74,84 ± 3,12 | 37,25 ± 2,5 | 4,02 ± 0,4 |
|  |  |  |  | N | 80,17 ± 12,38 | 54,48 ± 8,1 | 25,7 ± 4,39 | 68,75 ± 1,33 | 31,25 ± 1,33 | 57,29 ± 3,5 | 4,36 ± 0,35 | 663,23 ± 103,31 | 73,49 ± 3,27 | 27,63 ± 2,24 | 2,83 ± 0,25 |
|  |  |  | M | K | 38,7 ± 4,48 | 26,28 ± 4,01 | 12,42 ± 1,24 | 66,31 ± 2,71 | 33,69 ± 2,71 | 43,66 ± 4,81 | 5,48 ± 0,64 | 506,69 ± 51,54 | 76,84 ± 1,72 | 41,34 ± 2,72 | 4,54 ± 0,39 |
|  |  |  |  | N | 59,39 ± 5,82 | 41,63 ± 4,18 | 17,76 ± 2,17 | 69,79 ± 2,51 | 30,21 ± 2,51 | 55,85 ± 4,92 | 4,83 ± 0,56 | 513,89 ± 70,17 | 69,26 ± 3,06 | 29,28 ± 2,14 | 2,84 ± 0,31 |
|  |  | Winter | F | K | 33,89 ± 3,61 | 21,06 ± 1,97 | 12,83 ± 1,91 | 62,99 ± 2,4 | 37,01 ± 2,4 | 40,8 ± 3,38 | 6,04 ± 0,71 | 297,46 ± 50,82 | 59,24 ± 5,32 | 23,14 ± 1,7 | 1,94 ± 0,25 |
|  |  |  |  | N | 88,26 ± 4,83 | 57,67 ± 2,97 | 30,59 ± 2,64 | 65,68 ± 1,99 | 34,32 ± 1,99 | 47,33 ± 2,66 | 4,14 ± 0,18 | 709,67 ± 65,44 | 77,19 ± 1,05 | 24,37 ± 2,59 | 2,63 ± 0,33 |
|  |  |  | M | K | 38,54 ± 3,92 | 24,47 ± 2,54 | 14,07 ± 2,02 | 63,42 ± 3,61 | 36,58 ± 3,61 | 47,45 ± 5,94 | 5,15 ± 0,63 | 392,39 ± 75,72 | 65,22 ± 5,54 | 27,23 ± 2,12 | 2,59 ± 0,36 |
|  |  |  |  | N | 73,43 ± 6,37 | 52,33 ± 5,04 | 21,1 ± 1,75 | 70,49 ± 2,02 | 29,51 ± 2,02 | 54,42 ± 4,22 | 4,98 ± 0,78 | 410,43 ± 41,42 | 64,83 ± 3,31 | 19,39 ± 1,12 | 1,76 ± 0,16 |
| *Juniperus communis* L. | I | Spring | F | K | 14,94 ± 1,367 | 9,6 ± 0,82 | 5,34 ± 0,73 | 65,11 ± 3,16 | 34,89 ± 3,16 | 11,03 ± 0,7 | 5,64 ± 0,08 | 279,6 ± 41,08 | 92,09 ± 0,88 | 51,66 ± 0,99 | 10,23 ± 0,3 |
|  |  |  |  | N | 13,5 ± 1,471 | 8,93 ± 1,06 | 4,57 ± 0,48 | 65,82 ± 1,54 | 34,18 ± 1,54 | 11,04 ± 0,46 | 5,96 ± 0,2 | 239,99 ± 27,3 | 91 ± 0,62 | 52 ± 0,62 | 9,08 ± 0,16 |
|  |  |  | M | K | 11,81 ± 1,296 | 7,53 ± 0,81 | 4,27 ± 0,66 | 64,07 ± 2,85 | 35,93 ± 2,85 | 11,17 ± 0,63 | 5,92 ± 0,35 | 220,1 ± 37,62 | 92,3 ± 0,64 | 50,8 ± 0,75 | 10,15 ± 0,17 |
|  |  |  |  | N | 16,66 ± 1,384 | 11,03 ± 0,92 | 5,62 ± 0,62 | 66,68 ± 2,43 | 33,32 ± 2,43 | 11,96 ± 0,43 | 6,16 ± 0,22 | 300,63 ± 35,86 | 89 ± 0,73 | 52,91 ± 0,62 | 9,11 ± 0,23 |
|  |  | Summer | F | K | 16,38 ± 2,443 | 11,67 ± 1,52 | 4,71 ± 0,95 | 73,04 ± 1,71 | 26,96 ± 1,71 | 13,18 ± 0,59 | 6,48 ± 0,24 | 222,78 ± 31,01 | 86 ± 1,38 | 51,72 ± 3,02 | 8,49 ± 0,63 |
|  |  |  |  | N | 34,45 ± 5,045 | 27,38 ± 3,87 | 7,07 ± 1,29 | 80,09 ± 1,23 | 19,91 ± 1,23 | 12,93 ± 0,43 | 6,35 ± 0,25 | 380,88 ± 71,49 | 80,27 ± 2,07 | 52,48 ± 2,31 | 7,28 ± 0,54 |
|  |  |  | M | K | 11,86 ± 1,487 | 8,69 ± 0,86 | 3,53 ± 0,64 | 71,53 ± 1,89 | 28,47 ± 1,89 | 11,06 ± 0,76 | 7,63 ± 0,26 | 228,42 ± 43,46 | 88,99 ± 0,96 | 59,06 ± 4,09 | 10,28 ± 0,74 |
|  |  |  |  | N | 34,1 ± 3,356 | 26,64 ± 2,41 | 7,46 ± 1,01 | 78,79 ± 1,04 | 21,21 ± 1,04 | 10,63 ± 0,37 | 7,92 ± 0,26 | 443,06 ± 61,49 | 79,84 ± 1,36 | 59,73 ± 2,38 | 8,21 ± 0,49 |
|  |  | Autumn | F | K | 22,67 ± 2,44 | 15,16 ± 1,59 | 7,5 ± 0,92 | 67,28 ± 1,61 | 32,72 ± 1,61 | 13,66 ± 0,59 | 7,47 ± 0,25 | 352,45 ± 43,75 | 81,06 ± 1,46 | 49,88 ± 5,61 | 7,57 ± 0,86 |
|  |  |  |  | N | 63,89 ± 5,088 | 47,18 ± 3,85 | 16,71 ± 1,46 | 73,96 ± 1,12 | 26,04 ± 1,12 | 13,77 ± 0,57 | 7,19 ± 0,22 | 770,83 ± 95,85 | 75,12 ± 0,88 | 44,74 ± 3,25 | 5,65 ± 0,35 |
|  |  |  | M | K | 17,1 ± 1,912 | 11,14 ± 1,02 | 5,97 ± 0,93 | 66,56 ± 1,86 | 33,44 ± 1,86 | 12,26 ± 0,21 | 7,79 ± 0,25 | 289,48 ± 53,53 | 84,48 ± 2,57 | 47,54 ± 3,19 | 8,21 ± 0,86 |
|  |  |  |  | N | 53,67 ± 4,409 | 39,68 ± 2,64 | 13,99 ± 1,86 | 74,79 ± 1,45 | 25,21 ± 1,45 | 12,03 ± 0,29 | 8,21 ± 0,21 | 701,82 ± 121,72 | 82,35 ± 1,77 | 49,23 ± 3,37 | 7,31 ± 0,6 |
|  |  | Winter | F | K | 27,41 ± 2,856 | 17,31 ± 1,59 | 10,1 ± 1,3 | 64,42 ± 1,61 | 35,58 ± 1,61 | 13,74 ± 0,43 | 6,52 ± 0,18 | 391,09 ± 57,14 | 90,07 ± 1,95 | 43,11 ± 5,67 | 8,41 ± 1,21 |
|  |  |  |  | N | 81,54 ± 7,822 | 58,62 ± 5,31 | 22,91 ± 2,67 | 72,46 ± 1,16 | 27,54 ± 1,16 | 13,75 ± 0,62 | 6,92 ± 0,27 | 881,91 ± 114,51 | 87,31 ± 1,12 | 38,73 ± 2,56 | 6,05 ± 0,39 |
|  |  |  | M | K | 23,09 ± 2,312 | 14,46 ± 1,34 | 8,63 ± 1,05 | 63,25 ± 1,57 | 36,75 ± 1,57 | 13,49 ± 0,7 | 6,99 ± 0,19 | 389,31 ± 39,45 | 90,75 ± 0,56 | 46,94 ± 3,01 | 9,19 ± 0,68 |
|  |  |  |  | N | 75,57 ± 3,806 | 52,65 ± 3,02 | 22,93 ± 1,21 | 69,5 ± 1,19 | 30,5 ± 1,19 | 14,03 ± 0,75 | 6,82 ± 0,15 | 998,09 ± 67,72 | 86,45 ± 0,5 | 43,7 ± 2,28 | 6,62 ± 0,36 |
|  | II | Spring | F | K | 31,04 ± 2,503 | 20,74 ± 1,31 | 10,3 ± 1,23 | 68,02 ± 1,84 | 31,98 ± 1,84 | 13,33 ± 0,62 | 5,95 ± 0,13 | 433,8 ± 54,61 | 89,24 ± 0,96 | 42,17 ± 3,25 | 7,76 ± 0,74 |
|  |  |  |  | N | 97,24 ± 8,503 | 75,56 ± 6 | 21,69 ± 2,6 | 78,35 ± 0,99 | 21,65 ± 0,99 | 13,83 ± 0,49 | 6,57 ± 0,23 | 958,58 ± 132,6 | 88,79 ± 1,2 | 43,43 ± 2,23 | 7,1 ± 0,57 |
|  |  |  | M | K | 33,63 ± 1,025 | 21,79 ± 0,64 | 11,83 ± 0,56 | 64,91 ± 1 | 35,09 ± 1 | 12,94 ± 0,49 | 5,89 ± 0,14 | 518,57 ± 36,75 | 93,51 ± 0,65 | 43,98 ± 2,4 | 9,11 ± 0,61 |
|  |  |  |  | N | 89,03 ± 4,718 | 66,26 ± 3,58 | 22,78 ± 1,33 | 74,41 ± 0,65 | 25,59 ± 0,65 | 12,77 ± 0,66 | 6,7 ± 0,22 | 1053,85 ± 84,49 | 90,7 ± 0,84 | 46,06 ± 2,38 | 7,91 ± 0,51 |
|  |  | Summer | F | K | 43,62 ± 3,077 | 31,55 ± 2,01 | 12,08 ± 1,31 | 72,83 ± 1,67 | 27,17 ± 1,67 | 14,09 ± 0,41 | 7,52 ± 0,29 | 579,21 ± 57,31 | 89,42 ± 0,82 | 50,8 ± 5,74 | 8,93 ± 1,01 |
|  |  |  |  | N | 116,44 ± 16,74 | 94,75 ± 12,51 | 21,69 ± 4,47 | 82,6 ± 1,53 | 17,4 ± 1,53 | 13,18 ± 0,66 | 7,02 ± 0,33 | 1146,44 ± 237,63 | 88,37 ± 1,07 | 55,85 ± 6,19 | 9,34 ± 1,36 |
|  |  |  | M | K | 38,71 ± 2,483 | 26,4 ± 1,5 | 12,32 ± 1,02 | 68,45 ± 0,84 | 31,55 ± 0,84 | 14,67 ± 0,89 | 7,85 ± 0,4 | 638,55 ± 53,15 | 91,66 ± 0,7 | 51,84 ± 2,31 | 10,05 ± 0,54 |
|  |  |  |  | N | 149,38 ± 7,222 | 114,33 ± 5,46 | 35,05 ± 2,29 | 76,61 ± 0,87 | 23,39 ± 0,87 | 13,5 ± 0,76 | 7,04 ± 0,34 | 1837,1 ± 130,96 | 89,53 ± 1,02 | 52,77 ± 2,97 | 9 ± 0,8 |
|  |  | Autumn | F | K | 57,45 ± 2,993 | 41,19 ± 2,07 | 16,26 ± 1,32 | 71,87 ± 1,39 | 28,13 ± 1,39 | 13,48 ± 0,58 | 7,19 ± 0,17 | 546,56 ± 66,59 | 87,79 ± 1,69 | 32,78 ± 2,37 | 5,73 ± 0,57 |
|  |  |  |  | N | 143,67 ± 14,686 | 108,77 ± 10,82 | 34,9 ± 4,04 | 76 ± 0,79 | 24 ± 0,79 | 12,22 ± 0,32 | 6,63 ± 0,21 | 1367,95 ± 188,97 | 87,56 ± 1,16 | 38,82 ± 2,6 | 6,26 ± 0,51 |
|  |  |  | M | K | 60,12 ± 2,228 | 41,12 ± 1,38 | 19 ± 1,5 | 68,67 ± 1,67 | 31,33 ± 1,67 | 13,06 ± 0,64 | 6,97 ± 0,13 | 728,79 ± 63,61 | 90,88 ± 0,7 | 38,21 ± 2,01 | 7,13 ± 0,41 |
|  |  |  |  | N | 152,55 ± 10,005 | 115,87 ± 7,41 | 36,68 ± 3,26 | 76,13 ± 1,05 | 23,87 ± 1,05 | 12,63 ± 0,59 | 6,66 ± 0,17 | 1275,22 ± 95,11 | 88,36 ± 0,79 | 38,08 ± 2,98 | 6,43 ± 0,55 |
|  |  | Winter | F | K | 66,33 ± 6,055 | 43,93 ± 3,48 | 22,4 ± 2,71 | 67,44 ± 1,75 | 32,56 ± 1,75 | 12,82 ± 0,76 | 6,3 ± 0,31 | 768,3 ± 100,39 | 90,2 ± 1,4 | 33,44 ± 2,05 | 6,33 ± 0,48 |
|  |  |  |  | N | 173,88 ± 19,096 | 133,91 ± 14,72 | 39,98 ± 4,63 | 76,98 ± 0,73 | 23,02 ± 0,73 | 12 ± 0,62 | 5,93 ± 0,3 | 1368,05 ± 152,67 | 88,96 ± 1,02 | 34,95 ± 1,77 | 5,67 ± 0,24 |
|  |  |  | M | K | 64,81 ± 4,584 | 44,98 ± 3,35 | 19,83 ± 1,58 | 69,39 ± 1,28 | 30,61 ± 1,28 | 11,66 ± 0,7 | 6,37 ± 0,32 | 729,96 ± 69,37 | 90,99 ± 0,46 | 36,3 ± 1,15 | 6,93 ± 0,25 |
|  |  |  |  | N | 192,17 ± 9,33 | 144,99 ± 6,9 | 47,18 ± 2,54 | 75,49 ± 0,38 | 24,51 ± 0,38 | 11,6 ± 0,82 | 6,65 ± 0,31 | 1689,9 ± 182,03 | 88,82 ± 0,84 | 36,42 ± 3,74 | 6,26 ± 0,69 |
